# Mapping knowledge management resources of maternal, newborn and child health (MNCH) among people living in rural and urban settings of Ilorin, Nigeria

**DOI:** 10.11604/pamj.2014.17.34.2704

**Published:** 2014-01-20

**Authors:** Oladimeji Akeem Bolarinwa, Hafsat Abolore Ameen, Kabir Adekunle Durowade, Tanimola Makanjuola Akande

**Affiliations:** 1 Department of Epidemiology and Community Health University of Ilorin, Ilorin Nigeria; 2 Department of community medicine, Federal medical Center, Iddo - Ekiti Nigeria

**Keywords:** Maternal, child, newborn, knowledge management, mapping

## Abstract

**Introduction:**

Lack of access to information and knowledge about mother and child health was identified as a major contributor to poor maternal and child health in Nigeria. The Partnership for Maternal, Newborn and Child Health (PMNCH) has recognized mapping the knowledge management of Maternal Newborn and Child Health (MNCH) as one of the major strategies to be deployed in improving the health of these vulnerable groups. The main aim of this study is to map the knowledge management resources of Maternal, Newborn and Child Health (MNCH) in rural and urban settings of Ilorin West LGA of Kwara state Nigeria.

**Methods:**

It is a descriptive cross-sectional study with a comparative analysis of findings from urban and rural settings. Epi-mapping was used to carve out the LGA and map responses. The p-value of less than 0.05 was considered significant at 95% confidence level.

**Results:**

The study showed that traditional leader was responsible for more than half of the traditional way of obtaining information by rural (66.7%) and urban (56.2%) respondents while documentation accounts for the main MNCH knowledge preservation for the rural (40.6%) and the urban (50%) dwellers. Traditional leaders (32.2%) and elders (46.7%) were the main people responsible for dissemination of knowledge in rural areas whereas elders (35.9%) and Parents (19.9%) were the main people responsible in urban areas.

**Conclusion:**

It was concluded that traditional and family institutions are important in the knowledge management of MNCH in both rural and urban settings of Nigeria.

## Introduction

The importance and travail of mother and child was shown right from the creation of man and while mother and child status were been relegated to household materials their health status began to depreciate as well. Europe was the first to identify and address the vulnerability of women and children. Developing countries were carried along in the twentieth century when more international, local and non governmental agencies began to address the challenges of the mother and child [1]. The MDG declaration at the end of 20th century by the heads of state of UN countries made health issues of mother and child the 4^th^ and 5^th^ goals to stress the importance and vulnerability of the two groups [2]. In Nigeria the health situations of mother and child are one of the worst in the world, aside the major causes of maternal, newborn and child death in Nigeria, there are reinforcing risk factors that further worsen the health and survival rate of women and children, parts of which are poverty, low level of women education and gender inequalities, inadequate coverage and low quality of essential obstetric care in the country [2]. Lack of access to information in all forms that will cause delays of women to access quality health care is an important reinforcing factor that MNCH attentions has not been paid to over the years. This has also prevented adequate knowledge of the communities to the care of mother, newborn and child.

For instance majority of newborn (66%) are delivered by unskilled birth attendants at home by those who have poor knowledge of how to manage newborn condition 2 while only 40% of pregnant women in the country received two doses of tetanus toxoid required to protect them and their babies. Only 32% of the babies are initiated on breastfeeding within an hour of birth as required [2], while only 17% continued to be fed exclusively on breast milk for the first six months of life, whereas all of these are due to inadequate knowledge of the risks associated with these practices. Because these various factors are related to knowledge gap, therefore there is need to consider the Knowledge Management Systems (KMS) of the people in order to understand how knowledge could be created, stored and disseminated and how to integrate this strategy into Integrated Maternal Newborn and Child Health Strategies (IMNCH). Knowledge Management System (KMS) offers integrated services to deploy information instruments for networks of communities. KMS can be used for a wide range of cooperative, collaborative, adhocracy and hierarchy communities [3, 4].

The Partnership for Maternal, Newborn and Child Health (PMNCH) was launched in September 2005 as merger of pre- existing partnerships, with a focus on continuum care of MNCH and a main aim to accelerate the achievements of MDGs 4 and 5. It has 6 constituency groups; Developing country governments, Donors (bilateral and foundations), UN agencies (WHO, UNICEF, UNFPA, World Bank), Health care professional associations, Academic / training / research institutions and Non Governmental Organizations [3]. The strategic framework of PMNCH sets out six main inter-linked areas of work. The first of these, knowledge management, will establish a comprehensive knowledge management system which supports the creation, capture, storage and dissemination of information on MNCH, in order to provide readily-available robust knowledge summaries in a variety of formats, and to identify and flag critical knowledge gaps. The knowledge management system will include a web-based managed portal [3]. For the IMNCH interventions to be generally acceptable there would be need to inform and educate the masses on the need to change their behaviours and practices and this could only be possible if the Communities’ general knowledge acquisition, storage, sharing and transfer are identified. There would be the need to map these knowledge resources, identify and institute effective ways to utilize this for the purpose of managing the knowledge resources for Maternal, Newborn and Child Health interventions in the Country [3].

This new concept that is recently just gaining more recognition in health issues has been made use extensively in the last decade by commercial industries and organizations to preserve the organizational information, methods and technology [5–7]. It is mostly important in sharing of good practice and interventions on health and the concept of Community of Practice (CoP) is gaining more recognition everyday [8–10]. This is a concept that if adequately utilized could be employed to drive many health interventions, for community acceptability and ownerships of health programs like IMNCH in Nigeria and importantly in the aspect of Social marketing of reproductive commodities and MNCH care services [3, 4].

The poor health situation of mother and child health, led to a paradigm shift in 2007 to Integrated Maternal Newborn and Child Health (IMNCH), changing the thinking, orientation and practice to foster a continuum of care that will ensure survival of mothers and the children and hence future prosperity of the nation. There would be evidence based interventions that would be simple, cost effective and delivered to mothers and children who need them [2], some of which are already being implemented in other countries like Ghana, Mozambique, Tanzania, Uganda, Eritrea, Mali and Malawi with considerable improvements in their Maternal, Newborn and Child Health [2].

The rural and urban settings of the Country is important because of the difference in the aspects of populations since quite a large proportion of Nigerian population (70%) live in rural setting [11]. There are also differences in inequalities in access to health care, availability of infrastructure for knowledge resources, literacy levels, socioeconomic levels, perceived needs for Maternal, Newborn and Child Health, Cultural and traditional values variations, acculturations and varying levels of Maternal, Newborn and Child Health (MNCH) burdens. Therefore mapping the knowledge management resources of the rural and urban would help the decision makers, researchers and health workers to use the information in driving the Maternal, Newborn and Child health interventions for proper community acceptance, effectiveness of the programs therein and for a sustainable program. The main aim of this study however is to map the knowledge management resources of Maternal, Newborn and Child Health (MNCH) among the rural and urban settings of Ilorin West LGA of kwara state Nigeria.

## Methods

The study was a cross-sectional study with comparative analysis of the observed pattern between the rural and the urban clusters of adult populations in Ilorin, a North-central state of Nigeria. There was a household enumeration of the selected urban and rural communities. Household survey was carried out using a pretested semi structured questionnaire. The data was collected by interviewing a household head or most informed person per household. The minimum sample size for the rural and urban cluster was calculated using the formula for comparison of two proportions (comparing the rural with the urban respondents) [12]. Multi stage random sampling method was used to select respondents in the rural and urban communities.

For the purpose of this study a community with population of over 20,000 and presence of some of electricity supply, tarred road, Information technology (IT) facilities, industries and commercial firms, secondary and tertiary educational institutions and media houses were regarded as urban community. Analysis was done using Epi-info software package while mapping of knowledge management resources of the 2 settings was created using Epi-mapping of World Health Organization (WHO) to locate and carve out Ilorin west LGA from the State. The simulated map of Ilorin west LGA was carved so as to indicate the major areas of MNCH knowledge management like sources of general knowledge acquisition, main people responsible for Maternal Newborn and Child Health (MNCH) knowledge management in the communities and description of how respondents would manage MNCH knowledge in terms of storage and transfer. The p-value of less than 0.05 was considered significant at 95% confidence level.

## Results

**Figure 1:**
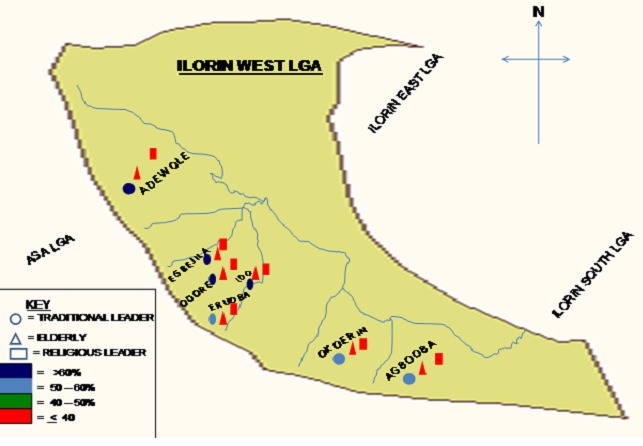
Map showing cultural ways of obtaining MNCH knowledge in rural and urban clusters

The age distribution of rural location is skewed towards elderly age group (44.1%) while the urban population was predominantly young adults (34.6%) and middle age (35.4%), with a p value of 0.00023169 (Table 1). There was no significant difference between the sex distribution of the two locations (p=0.65866). There was however difference in marital status of the respondents in the rural and urban areas (p=0.00000234) with more unmarried respondents in the urban (25.4%) than the rural (1.8%) areas. There was also significant difference (p=0.00000000001) in the literacy level of both areas with over 80% of illiteracy level observed in the rural area as opposed to 41.5% illiteracy level in the urban area. Traditional leader through the traditional town announcer was responsible for more than half of the traditional way of obtaining information by rural (66.7%) and urban (56.2%) respondents. There is no difference in the traditional route of obtaining information in both areas (p=0.13623331). Other routes recognized by the study as sources of obtaining general health information in both settings were through elderly members, religious routes and other family sources (Figure 1).

**Table 1:** Socio-demographic distribution of the respondents

VARIABLE	Rural (%) (N=111)	Urban (%) (N=130)
**Age distribution**		
< 21	0 (0)	8 (6.2)
21 – 30	7 (6.4)	26 (20)
31 – 40	11 (9.9)	19 (14.6)
41 – 50	24 (21.6)	26 (20)
51 – 60	20 (18)	20 (15.4)
> 60	49 (44.1)	31 (23.8)
	X^2^ = 23.85, df = 5	p = 0.00023169
**Sex distribution**		
Male	43 (38.7)	54 (41.5)
Female	68 (61.3)	76 (58.5)
	X^2^ = 0.20, df = 1,	p = 0.65866
**Level of education**		
None	70 (63.1)	29 (22.3)
Primary	26 (23.4)	25 (19.2)
Secondary	14 (12.6)	32 (24.6)
Tertiary	1 (0.9)	44 (33.9)
	X^2^ = 63.03, df = 3	p = 0.0000001
**Distribution by cluster of communities used for the study**		
**Rural Communities**		**Urban Communities**
Egbejila	35 (31.5)	Adewole: 42 (32.3)
Eruoba	24 (21.7)	Agbooba: 40 (30.8)
Ido	26 (23.4)	Okoerin: 48 (36.9)
Odoore	26 (23.4)	

Traditional leaders (35.9%) and Elders (41.8%) played major roles in knowledge storage in the rural areas while Elders (35.3%), Parents (17.8%) and head of family (15.1%) were the main people responsible for knowledge storage in urban areas. This difference was significant (p=0.000000001). Similar pattern existed for the dissemination of health knowledge. Traditional leaders (32.2%) and Elders (46.7%) are the main people responsible for dissemination of knowledge in rural areas whereas Elders (35.9%) and Parents (19.9%) were the main people responsible in urban areas.

**Figure 2:**
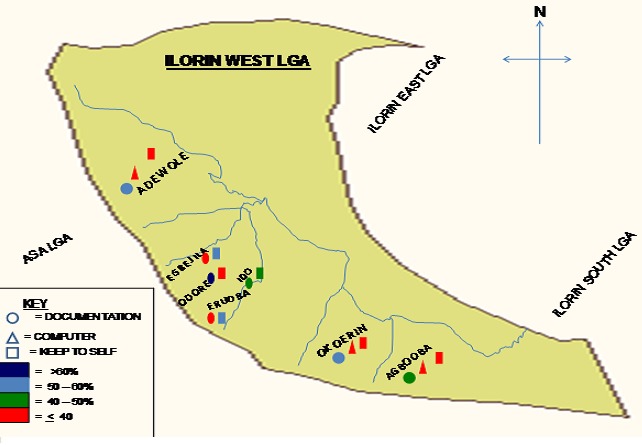
Map showing how MNCH knowledge is being preserved in rural and urban clusters

Traditional leaders are the main cultural ways of obtaining MNCH knowledge in all the rural and urban clusters with no significant difference in all the clusters (Figure 1). Documentation accounts for the main MNCH knowledge preservation for the rural (40.6%) and the urban (50%) dwellers (Figure 2), but significant proportion of the urban dwellers (17.7%) would use Computer and other Information technology (IT).

**Figure 3:**
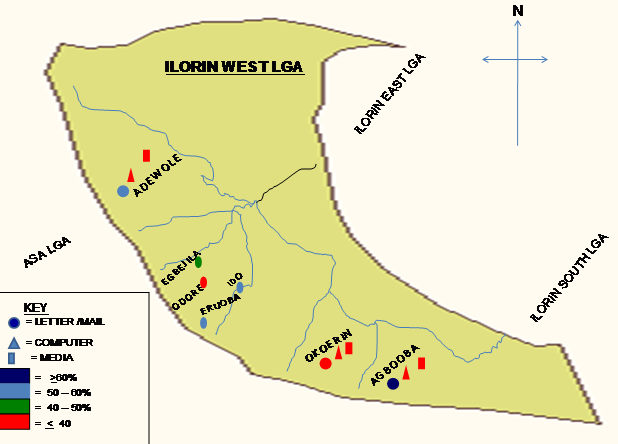
Map showing how knowledge is being disseminated in rural and urban clusters

Letter writing/Mailing is responsible for almost half of the medium that respondents in rural (43.2%) and urban (48.4%) areas would use to disseminate MNCH knowledge. However, Computer and other IT media is significantly higher in urban (18.5%) than rural (3.6%) area. All the 4 clusters in the rural area would like to disseminate MNCH knowledge through mail and letter writing while respondents in the 3 clusters in urban area would like to disseminate MNCH knowledge mostly through letter writing or mailing and computer (Figure 3). More urban (80.8%) respondents had knowledge of Information Technology (IT) as compared to their rural (63.1%) counterpart. This difference is significant with p-value of 0.0021236. However, more than half of the respondents that claimed knowledge of Information technology could not correctly described it (p=0.615112). Only 4.5% of rural respondents had access to computer/internet facilities as opposed to 70% of the urban respondents. There were high usage rates among those respondents in the rural (60%) and urban (91.9%) area that have the access.

There is high preference for modern mode of MNCH knowledge management across all levels of education among respondents in both rural (p = 0.7636) and urban (p=0.0974) areas. All the unmarried respondents in rural setting have no knowledge of health problems of mothers and children while 42.4% of those in urban setting had no knowledge of health problems of mothers and children. These observations are significantly different in both settings (p<0.005). Twenty five percent of Artisans in rural area and 41.2% in urban area have no knowledge management of MNCH as compared to other occupations. These observations were significantly different in the rural (p=0.0133) and urban (p=0.001) settings.

## Discussion

The 61% of female in rural and 58.5% of female in urban were similar to response pattern observed in Nigeria demographic and health Surveys (NDHS) of 2008 [13] where 68% and 65% were proportions of female respondents in the rural and urban settings respectively. Expectedly, while over 60% of the rural respondents were illiterates, only 22.3% of the urban respondents were illiterates. One third of both the rural and urban respondents were traders but the major difference was that while Farmers and Artisans were more than another third of rural respondents, Civil servants and students constitute more than one-third of urban respondents.

This study showed the relative knowledge management difference between the rural and urban preferences on a simulated map of Ilorin west LGA of kwara state, so as to indicate the major areas of knowledge management where the main differences lied like sources of general knowledge acquisition, main people responsible for knowledge management in the communities and description of how respondents would manage knowledge in terms of storage and transfer. This representation is necessary so as to be able to show at a glance the knowledge management preferences of both settings for evidence based decision making which is the hallmark of IMNCH strategy [14].

More than half of the respondents from the rural and urban settings obtained their knowledge traditionally through traditional leader. This involved the use of “Town criers“ now known as “Town announcer“ who relates information from the traditional leader to his subjects. The role of traditional institution in Nigeria has been severally emphasized in Health programs especially in community participation and community ownership of health programs [15]. The traditional institution has culturally made use of town announcers to spread announcements on health programs and this has been extensively used in Immunizations [16]. The implication of this findings is that Knowledge management of IMNCH in Nigeria should take into consideration active involvement of traditional institution in Nigeria in storage and transfer of knowledge on IMNCH for the strategy to have success. Other traditional ways that should be considered according to the findings from this study are; the role of elderly people in the community, religious institutions and the use of tales and music to manage knowledge resources on IMNCH.

The difference between the main people responsible for knowledge storage in the rural and urban settings in this study implies that while traditional institutions could be used to store knowledge on IMNCH in the rural communities it would be more effective to use elders and family institution in the urban setting and this also encourages the tacit knowledge storage [17] and subsequent transfer through coaching and mentoring [18]. Similarly, this study revealed that there is a difference in the particular persons responsible for knowledge dissemination in rural and urban settings with a p-value of 0.0000001. Again traditional leaders and elders were the main people in rural settings while Elders, parents and head of family were the main people in the urban settings. However, other relevant categories of people that are responsible for knowledge dissemination in the two settings are; political leaders and opinion leader.

Documentation by writing the knowledge down in a secured medium like books, diaries, audio recording and video recording were the most common description of how the respondents in rural and urban areas would like to store knowledge. The main significant difference in the two areas was, while only few of the rural respondents would use Information Technology (IT) to store knowledge, some significant proportion of urban respondents would use IT. These findings implied that documentation in terms of keeping diaries and media recording is important in knowledge storage and should be taken into consideration while determining the knowledge management of IMNCH strategies. Many of the evidence based interventions [14] that are required to change the trends of maternal, newborn and under fives morbidity and mortality required proper documentation in form of audio visual recordings and literary as a form of Community of Practice (CoP) [8–10].

When compared, IT usage is more pronounced in urban settings than the rural setting because of social amenities and infrastructural availability [9, 19]. But notably, among the urban respondents, only few would like to store knowledge through IT. This finding is worrisome because web-based managed portal is the main strategy that the knowledge management of MNCH is intended to use [3] and while this could be fairly successful in urban settings the use in the rural setting may not be effective.

It was shown that the preferences of knowledge management for MNCH are affected to a small extent by rural and urban settings. This was evident from the fact that there is no statistical significance in the observed traditional ways of knowledge acquisition in both settings, their preferences for traditional and modern knowledge management for MNCH and general perceptions to knowledge management of MNCH in the two settings. However, the effect was more in the area of sources of general knowledge acquisition, main people responsible for knowledge management, access and usage of IT and awareness and knowledge of the mother and child health problems.

## Conclusion

This study concluded that traditional and family institutions were important in knowledge management of maternal and child health in both the rural and urban settings of Nigeria while IT is a potential source of strength for knowledge management of MNCH. It is recommended that there is need to accommodate the traditional institution in the knowledge management of MNCH by the IMNCH Core Technical Committee (CTC) of both Federal Ministry of Health (FMoH) and State Ministry of Health (SMoH). The health policy makers at all the 3 tiers of government should endeavor to explore the traditional and family routes of knowledge management to preserve good health practices as it relates to mother and child.
